# Estimation of Andrographolides and Gradation of *Andrographis paniculata* Leaves Using Near Infrared Spectroscopy Together With Support Vector Machine

**DOI:** 10.3389/fphar.2021.629833

**Published:** 2021-05-06

**Authors:** Dilip Sing, Subhadip Banerjee, Shibu Narayan Jana, Ranajoy Mallik, Sudarshana Ghosh Dastidar, Kalyan Majumdar, Amitabha Bandyopadhyay, Rajib Bandyopadhyay, Pulok K. Mukherjee

**Affiliations:** ^1^ Department of Instrumentation and Electronics Engineering, Jadavpur University, Salt Lake Campus, Kolkata, India; ^2^ School of Natural Product Studies, Jadavpur University, Kolkata, India; ^3^ Institute of Bioresources and Sustainable Development, Imphal, India

**Keywords:** near infrared spectroscopy, minimal processing, medicinal plant, Andrographis paniculata, quality grading, support vector machine

## Abstract

*Andrographis paniculata* (Burm. F) Nees, has been widely used for upper respiratory tract and several other diseases and general immunity for a historically long time in countries like India, China, Thailand, Japan, and Malaysia. The vegetative productivity and quality with respect to pharmaceutical properties of *Andrographis paniculata* varies considerably across production, ecologies, and genotypes. Thus, a field deployable instrument, which can quickly assess the quality of the plant material with minimal processing, would be of great use to the medicinal plant industry by reducing waste, and quality grading and assurance. In this paper, the potential of near infrared reflectance spectroscopy (NIR) was to estimate the major group active molecules, the andrographolides in *Andrographis paniculata*, from dried leaf samples and leaf methanol extracts and grade the plant samples from different sources. The calibration model was developed first on the NIR spectra obtained from the methanol extracts of the samples as a proof of concept and then the raw ground samples were estimated for gradation. To grade the samples into three classes: good, medium and poor, a model based on a machine learning algorithm - support vector machine (SVM) on NIR spectra was built. The tenfold classification results of the model had an accuracy of 83% using standard normal variate (SNV) preprocessing.

## Introduction

*Andrographis paniculata* (Burm. F) Nees, popularly known as the “king of bitters,” is an herbaceous plant in the family Acanthaceae. In China, India, Thailand, and Malaysia, this plant has been widely used for treating sore throat, flu, and upper respiratory tract infections (Jayakumar et al., 2013). Its major constituents are the group of diterpenoids designated as andrographolides. Andrographolides have a broad range of therapeutic activities, such as anti-inflammatory (Shen et al., 2002), antibacterial, antitumor, antidiabetic, antimalarial, and hepatoprotective (Trivedi and Rawal, 2001; Jayakumar et al., 2013; Dai et al., 2019). Clinical trials suggest that *Andrographis paniculata* has a strong treating capacity of viral respiratory infections (Hu et al., 2018).

The genotype and agroecology of *Andrographis paniculata* determine the nature and quantity of the active molecules like andrographolides and hence the pharmacological activities (Jayakumar et al., 2013). It is important to estimate the most important active molecules, the andrographolides and classify *Andrographis paniculata* plants with respect to this group of marker molecule. Traditional classifications based on visual inspection of the morphological traits do not consider the active molecules at all. Thus, estimations of active molecules for pharmacognosy, physicochemical studies are mainly based on analytical techniques like high performance liquid chromatography (HPLC), high performance thin layer chromatography (HPTLC), LC-MS/MS (Mukherjee et al., 2015). These techniques are time-consuming, complicated, labor-intensive, and expensive; moreover, they produce considerable quantities of wastes (Borraz-Martínez et al., 2019). Any field level quality control system should be able to handle a large number of samples fast, at a relatively low cost and reasonably dependable accuracy and precision.

Use of NIR Spectra for detection and estimation of specific chemical constituents has proved its worth in diverse fields of application. Its advantages are low cost, speed and simplicity of detection, need for minimal sample preparation and even nondestructive use of samples, feasibility of use at the field level and the feasibility of mechanizing the detection/estimation system for large number of samples. NIR spectroscopy uses liquid and solid samples without any pretreatment. Even when sample extracts are used, NIR spectroscopy obviates the use of further costly chemicals, equipment and time that are required in standard techniques like HPLC. NIR spectroscopy has been successfully used in several areas, like, in the tea leaf quality assessment (Hazarika et al., 2018), in food safety assessment (Cen and He, 2007; Lee and Herrman, 2016; Kusumaningrum et al., 2018) environmental chemistry, microfluidics, biomolecules (Cho et al., 2009), cancer diagnostic agents, and forensic science (Muehlethaler et al., 2016) as well as explosive detection.

In this study, the feasibility of using NIR spectra for estimating andrographolides in *Andrographis paniculata* plant samples and grading the samples based on the estimates was investigated. The performance of NIR spectrum-based technique was examined both in samples of dry powdered leaves and methanol extracts of leaves. Methanol extracts were required for estimation through HPLC, the gold standard (Mukherjee, 2019). If NIR spectrum can estimate andrographolides with reasonable accuracy compared to HPLC, that itself will be a big advantage in saving cost, time, and make mechanization of large scale estimates feasible.

We also attempted to grade the *Andrographis paniculata* samples based on the content of andrographolides (IP 2014). As only a small portion of the spectra responds to the andrographolides, a low cost portable spectrometer with customized range can be employed for simple gradation of the samples without the need for highly accurate estimation, and is likely to be used as a ready-to-apply tool by the traders for breeding and improvement of cultivars for obtaining higher drug yield (Raina et al., 2013).

## Materials and Methods

### Plant Material

Plant material (leaves) of *Andrographis paniculata* was collected from West Medinipur, East Medinipur, Purulia, South 24 Parganas, Hooghly, and Kolkata district of West Bengal, India. Plant material were identified and authenticated by Dr S. Rajan, Field botanist, the medicinal plant collection unit, Ooty, Tamil Nadu, Govt. of India. The voucher specimens of 18 samples (specimen no. SNPS/JU/2018/12–29) were deposited at School of Natural Product Studies, Jadavpur University, Kolkata, India for future reference.

Based on the content of andrographolides, *Andrographis paniculata* samples were graded into 3 categories: good quality, medium quality and poor quality. Plants having high andrographolides content (>2.50%) (Raina et al., 2013)were considered to be of good quality and placed in grade I; those with <1%, the non-acceptable level according to the Indian Pharmacopia (IP 2014), were placed in grade III. The intermediate types (≥2.5% and ≤1%) were placed in grade II.

### Chemical Analysis

#### Chemicals and Reagents

Andrographolides standard (>95% HPLC) was obtained from Sigma Aldrich. Methanol (HPLC grade), glacial acetic acid (HPLC grade), petroleum ether and ethyl acetate (analytical grade) and all other solvents (AR grade) were procured from Merck, India. Quantitative estimation was performed with Empowers software using the external standard calibration method.

#### Extraction

The air-dried (at room temperature) samples of 18 plants were powdered to a moderately coarse texture (180–355 µm) by a mechanical grinder. Methanolic extracts were made from the powdered samples (50 g in 80 ml of methanol) using rotary shaker at 150 rpm for 10 mins. Each sample was extracted thrice. The extracts were filtered and dried by vacuum evaporation using a rotary evaporator at 50°C and high pressure. The dried extract (10 mg) was dissolved in methanol and filtered through 0.22 µm membranes to get the stock solution (10 mg/ml). The stock solution was diluted to get 1 mg/ml sample concentration for HPLC.

#### High Performance Liquid Chromatography Analysis

**FIGURE 1:**
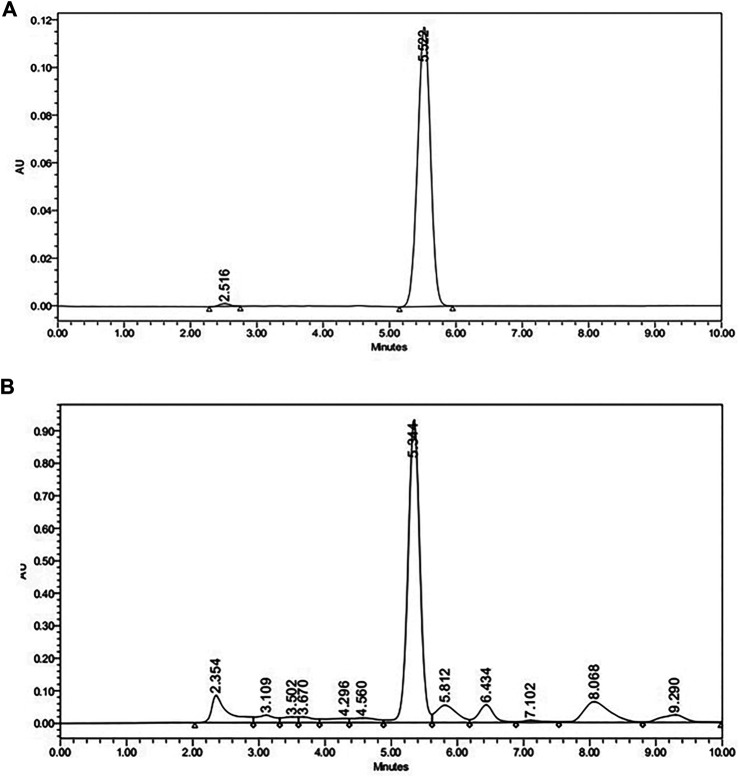
HPLC chromatogram of **(A)** Andragrapholides Standard **(B)** *Andrographis paniculata* extract sample.

The Reverse phase HPLC (RP-HPLC) system (Waters, United States of America) equipped with a rheodyne 7725i injector having 20 µL loop, 3 Lines degasser (Volume 400 μL), LC-30AD pump, C18 column (5 µm particle size, 250 × 4.6″) UV/Vis detector was used for the analysis. The method developed was isocratic with the mobile phase of Methanol: Water (1% acetic acid) - 60:40 v/v/v. A Milli-Q academic water purification system (Bedford, MA, United States of America) equipped with 0.22 μm Millipak express filter. The pH of the solvent B was adjusted at 2.4 by using 1% (v/v) glacial acetic acid for better ionization. The mobile phase was filtered through 0.45 mm pore size (Millipore) membrane filter followed by sonication to degas the solvent. Whatman syringe filters (NYL 0.45 μm) were used for the filtration of the sample. The temperature of the column was kept at 25°C and the injection volume was 20 µL. The total run time was set at 10 min. The flow rate was set at 1.0 ml/min and the *λ*_max_ was set at 230 nm for maximum absorption of the compound. Quantitative estimation was performed with Empower 2 software programs using the external calibration method. Typical chromatograms of andrographolides standard and an *Andrographis paniculata* extract sample are shown in Figure 1.

### Method Validation

The RP-HPLC method was validated on the basis of the International Conference on Harmonization guidelines (ICH Q2 (R1) Guideline) (ICH Harmonized Tripartite Guideline, 2005). Method specificity was determined by comparing the retention time of both the standard and test samples. Limit of Detection (LOD) and Limit of Quantification (LOQ) were calculated based on the equation: LOD = 3.3 *σ/S* and LOQ = 10 *σ/S*, where *σ* is the residual standard deviation of regression and *S* is the slope of the calibration curve. The accuracy of the method was determined as percent recovery by the assay of known added amount of analyte in the sample. The samples were spiked with three different concentrations of standard compounds in triplicate. The precision of the analytical method was assessed by measuring six replicates of each of three different concentrations of the reference compounds. The results were represented as %RSD of intra-day and inter-day analysis.

### Experimental Setup and Near Infrared Spectroscopy Spectra Acquisition

**FIGURE 2:**
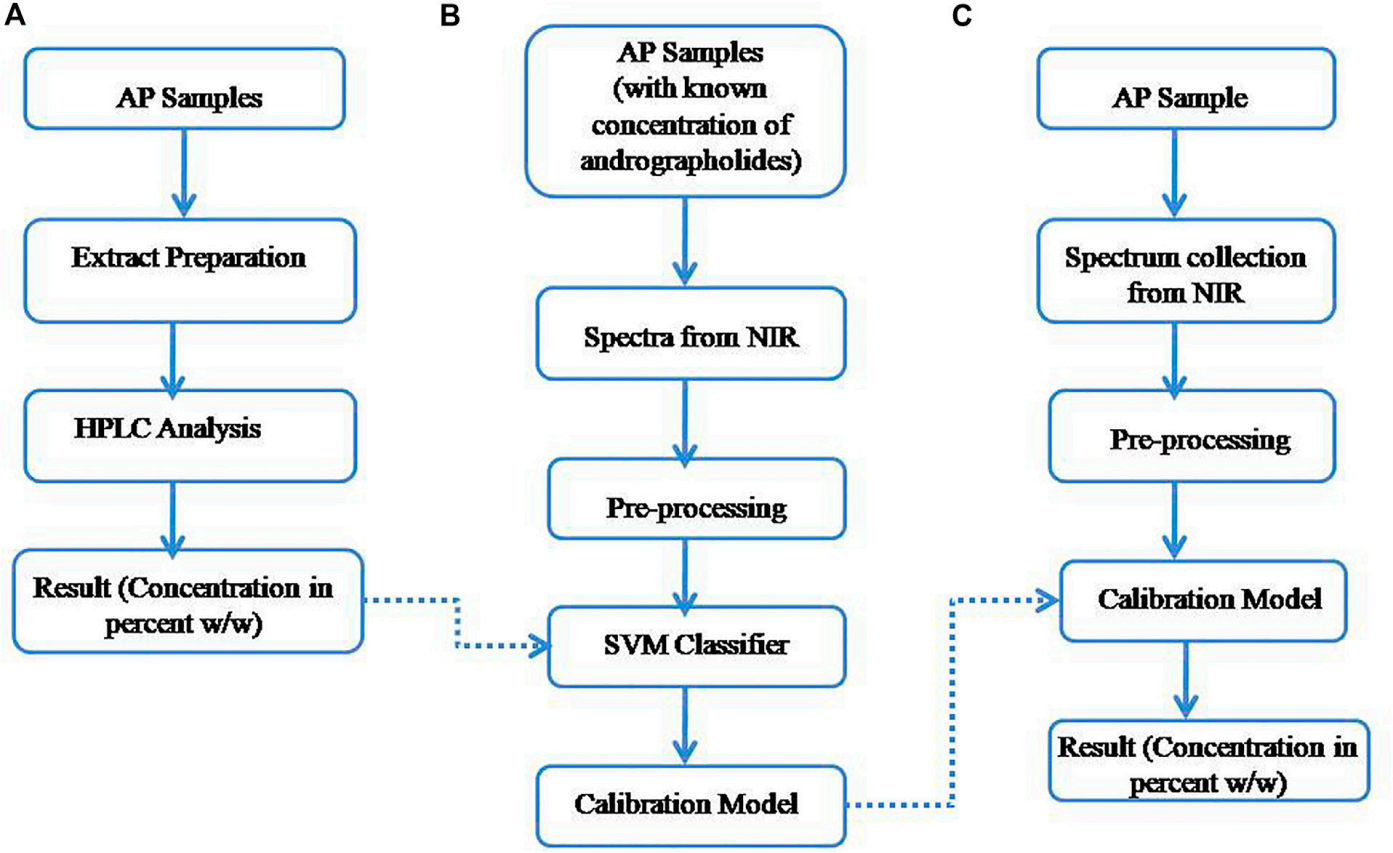
Experimental workflow of **(A)** chemical analysis **(B)** training, **(C)** testing.

A DWARF-Star NIR spectrometer (StellarNet Inc., United States of America) coupled with an upward looking diffuse reflectance accessory RFX-3D was used to acquire the diffuse reflectance spectra of the medicinal plant samples. The scanning range was from 900 to 1700 nm, and a RS50 (50 mm diameter White reflectance standard, halon>97%) was used as a calibration reference. The sample (after drying and grinding) was placed in a standard quartz plate of 1 mm thickness at the top of RFX-3D. RFX-3D was integrated with a 5 W halogen bulb and three fiber connectors to the spectrometer each positioned 120° in a circle to eliminate the need to rotate for coarse grain or non-uniform samples. Each sample was scanned 16 times with integration time of 300 ms and then the averaged spectrum was used for analysis. The S/N ratio was 4,000:1, wave-number accuracy was within ±0.01 cm^−1^, and the resolution was set as the resolving resolution of 2.5 nm. The temperature was kept at about 25°C during the whole experiment. Figure 2 explains the workflow of the chemical analysis, and the training, and testing steps with NIR spectrum.

In the Stellarnet spectrometer used in our study, the data points were collected from 900 to 1700.5 nm with a gap of 1.75 nm. Thus there were (1700.5–900)/1.75 = 459 points for each spectrum. Fifteen (15) replicates were taken for each sample; thus, in total, 459 × 18 × 15 spectral data points were obtained for 18 samples.

## Data Analysis

Both raw and pretreated spectral data obtained from the NIR instrument with the *Andrographis paniculata* samples were analyzed. To reduce the effect of scattering, data was pretreated with standard normal variate (SNV) method. The chemometric methods - principal component analysis (PCA), linear discriminant analysis (LDA) and support vector machine (SVM) were applied for clustering and classification.

### Preprocessing

In order to reduce the effect of base line variation, and scattering of light, standard normal variate (SNV) preprocesing technique was used on NIR spectra. The method removes the offset by subtracting the mean value of the full spectrum and brings all the spectra to the same scale dividing by the standard deviation of the spectrum (Barnes et al., 1989).

### Principal Component Analysis

Principal component analysis (PCA) (Pearson, 1901) was used in order to visualize the multivariate data, by transforming the data along orthogonal axes, and the first two axes were plotted to observe the discrimination between the samples. Here, each point in the PCA plot corresponds to one spectrum.

### Linear Discriminant Analysis

For reduction of features, Linear Discriminant Analysis (Fisher, 1936) was also used to visualize the samples in a lesser dimension hyper-plane. LDA maximizes separation between the samples. Only the first two axes were considered for visualizing the data. Class information was used to plot the data. PCA and LDA were implemented in Matlab V10.0 (Mathworks Co., United States of America).

### Support Vector Machine

Support vector machine (SVM) is a machine learning algorithm (Cortes and Vapnik, 1995). We have used radial basis function (RBF) as the kernel function. In this study, the two parameters of SVM - *γ* (RBF kernel width) and *c* (SVM cost factor) were obtained based on the minimal classification error through a two-dimension grid search coupled with a leave-ten-out cross validation.

Full-spectrum data was directly used as the input of SVM to build the gradation model. The search range for *γ* was set from 10^–6^ to 10 with 15 values spaced uniformly for *c* from 10^–3^ to 100 with 11 values spaced uniformly. The optimal parameter combination of *γ* and *c* were obtained as 0.0001 and 100, respectively.

Support vector machine (SVM) was implemented in R studio. The accuracy of classification was estimated on the basis of the tenfold cross-validation method, where nine subsets were used for training and one subset for testing.

## Results and Discussion

### Interpretation of Spectroscopic Characterization

#### Original and Preprocessed Spectra

**FIGURE 3:**
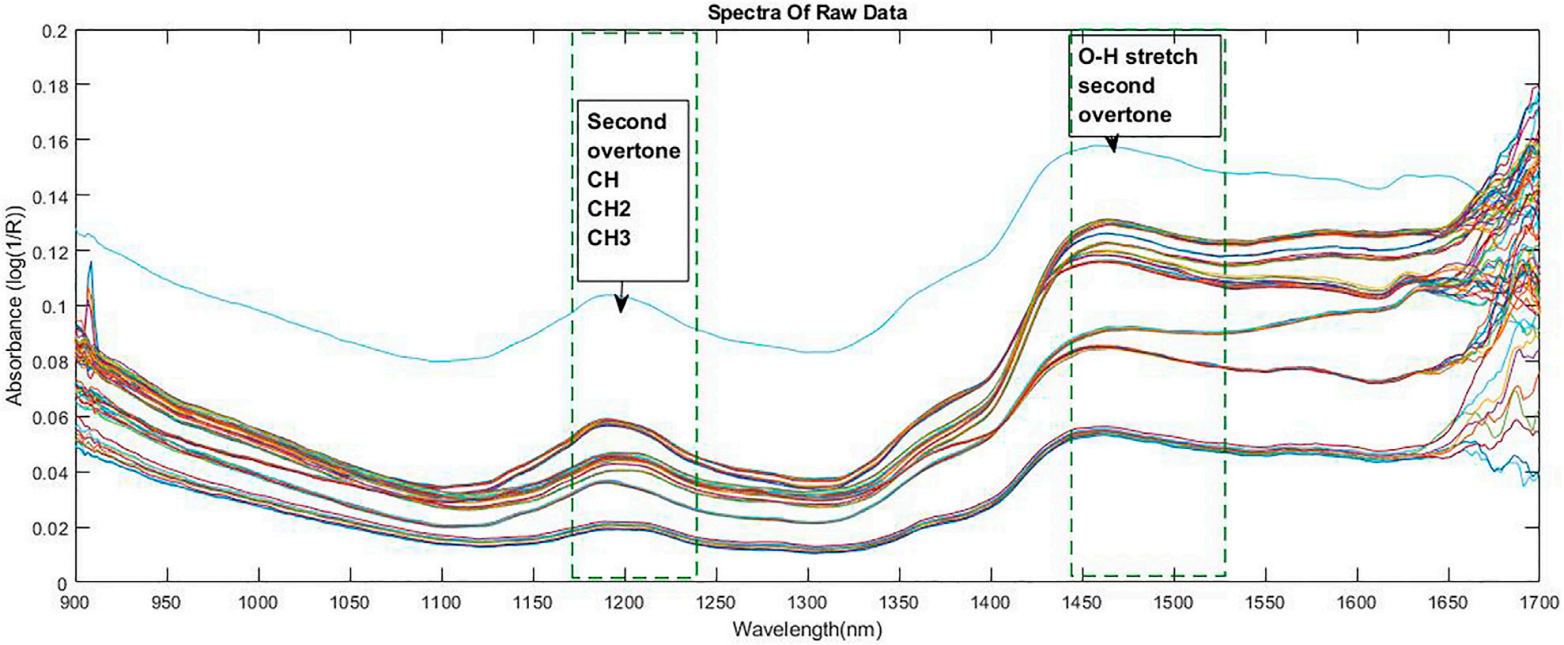
Wavelength vs. absorbance of the original spectra without pre-processing.

The graphical representation of the wavelength vs. absorbance of the raw spectra without any pre-processing is shown in Figure 3. The spectra obtained using the dried leaf powder shows absorption peaks at 1200 nm (C-H Second overtone region due to presence of CH2, CH) and 1472 nm (O-H second overtone region due to presence of R-OH group) (Reuben et al., 2018).

#### Loading Plot

**FIGURE 4:**
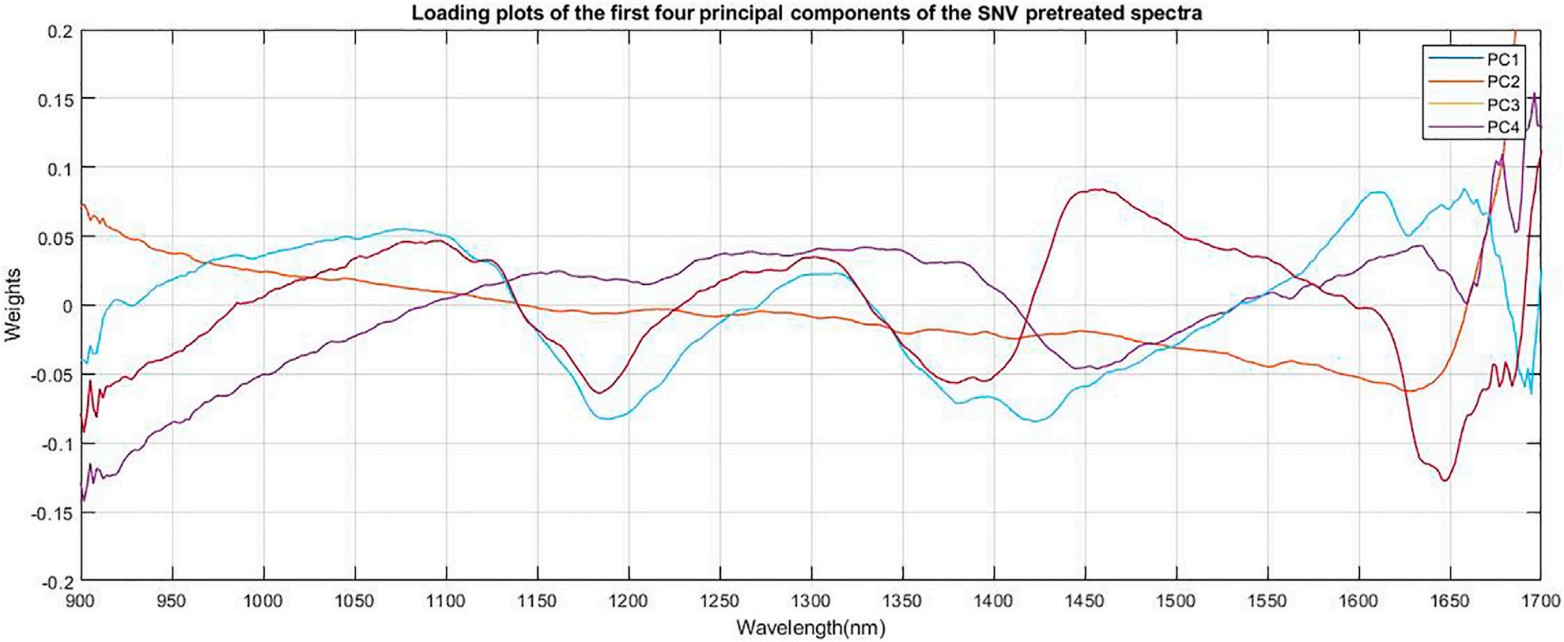
Loading plots of the first four principal components of the SNV pretreated spectra of dried *Andrographis paniculata* plant material.

The loading plot of the spectra is shown in Figure 4. The relative contributions of each PC to the explained variation were large for the first few components, with 97.2% of the variation explained by the first four components for andrographolides (PC1 = 55.89%, PC2 = 21.82%, PC3 = 15.94%, and PC4 = 3.56%).

**FIGURE 5:**
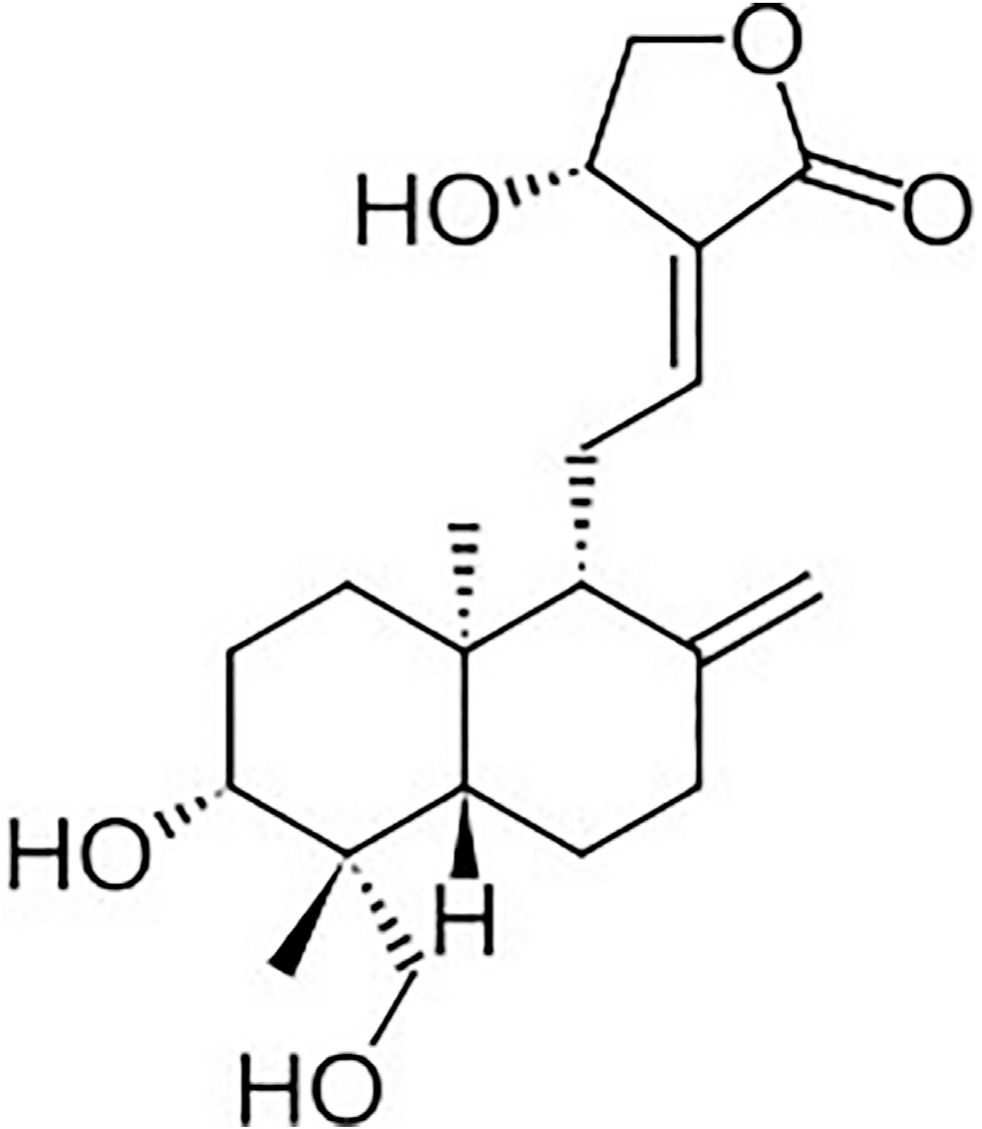
Chemical structure of andrographolides.

The first two principal components (PCs) showed strong absorption peaks at 1100 and 1300 nm (C-H Second overtone region due to presence of CH3). The loading plot of PC2 (Figure 4) a showed another absorption peak at 1450 nm (O-H Second overtone region due to presence of R-OH group) and loading plot of PC1 showed another absorption peak at 1650 nm (C-H first overtone region due to presence of CH3). The loading plot of PC4 (Figure 4) had major absorption peaks at 1400 nm (O-H second overtone, associated with water) and 1650 nm (C-H first overtone region due to presence of CH3, CH2, CH). These are all related to the most abundant structural groups present in the andrographolides (Figure 5).

### Plot of Principal Component Analysis

**FIGURE 6:**
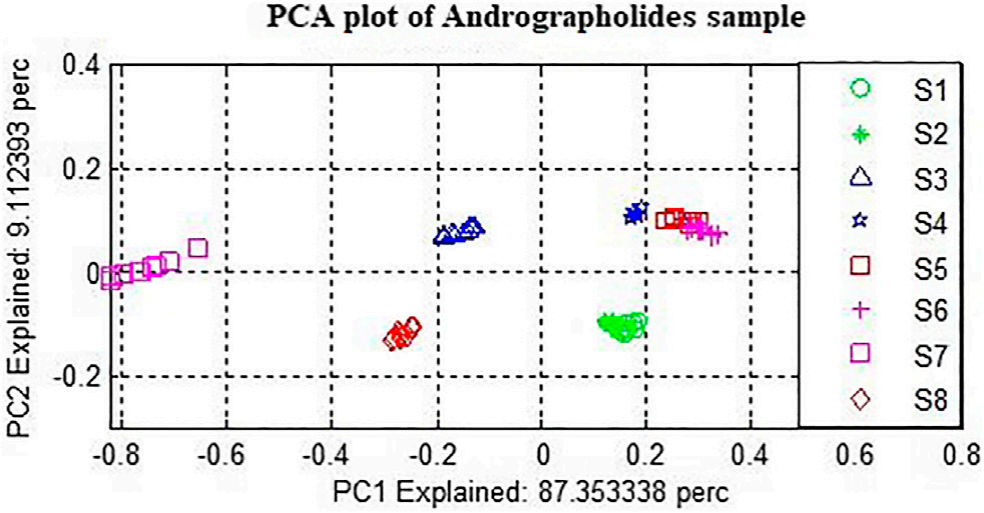
PCA plot with first two PC.

PCA was performed on the raw and preprocessed spectra to examine the qualitative differences among the three varieties of the samples. Figure 6 presents the PCA score plot derived from the raw spectra of *Andrographis paniculata* samples. The first and second principal components (PCs) accounted for 90.67% and 7.89% of the total variance, respectively. Samples of the same variety have been observed to appear as clusters, and no overlaps were observed among the three samples.

### Plot of Linear Discriminant Analysis

**FIGURE 7:**
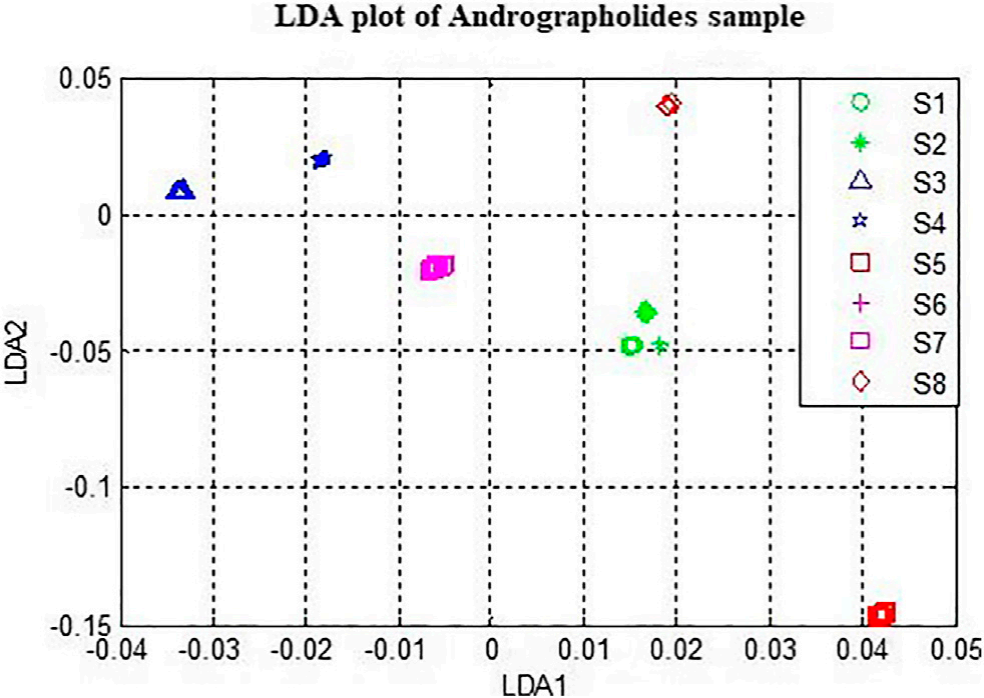
LDA plot with first two components.

Figure 7 displays the Linear Discrimination Analysis plot applied to the whole data matrix after preprocessing using Standard Normal Variate (SNV). The discrimination is better than PCA.

### Support Vector Machine Model

For analysis on the spectra obtained from the ethanol extracts of 18 samples, the support vector model was used for regression on the data pretreated with SNV data. The results of HPLC analysis, NIR predicted values and the corresponding grades are shown in Table 1. The of correlation coefficients and paired two tailed “*t*” statistic results of three pairs of among three sets of estimates: NIR estimates of methanol extract, NIR estimates of powdered samples, and the estimates from HPLC analysis, are given in Table 1.

**TABLE 1:** Result of HPLC and NIR and the grades of the 18 samples.

Serial no	Sample ID	HPLC method (w/w %)	Prediction by NIR		Grade
			Extract (w/w %)	Powder (w/w %)	
1	SNPS/JU/12	2.50	2.45	2.25	II
2	SNPS/JU/13	2.20	2.10	2.00	II
3	SNPS/JU/14	1.5	1.45	1.44	II
4	SNPS/JU/15	0.53	0.47	0.41	III
5	SNPS/JU/16	0.54	0.54	0.49	III
6	SNPS/JU/17	0.55	0.50	0.44	III
7	SNPS/JU/18	0.93	0.90	0.89	III
8	SNPS/JU/19	2.90	2.89	2.46	I
9	SNPS/JU/20	2.65	2.62	2.52	I
10	SNPS/JU/21	1.71	1.70	1.65	II
11	SNPS/JU/22	2.57	2.54	2.32	I
12	SNPS/JU/23	2.72	2.62	2.52	I
13	SNPS/JU/24	2.68	2.31	2.14	I
14	SNPS/JU/25	2.30	2.28	2.29	II
15	SNPS/JU/26	1.57	1.80	2.07	II
16	SNPS/JU/27	1.79	2.24	2.05	II
17	SNPS/JU/28	1.59	1.54	1.44	II
18	SNPS/JU/29	2.03	2.13	2.12	II
	Correlation coefficient with respect to HPLC results		0.97	0.94	
	“*t*” value		0.27	1.78	
	“*p*” value		0.79	0.09	

Eighteen data patterns were considered for the tenfold cross-validation method. In this method, a total of ten train-test trials were conducted, and the total data set was divided into ten subsets. Out of these ten subsets, one subset (10% of data) was used as the test set, and the other nine subsets (90% of data) were used as the training set. The classification rates were then averaged over these folds for estimating the classifier performance. The classification rate and the total misclassified pattern on 18 NIR data are shown in Table 2.

**TABLE 2:** Result of 10-fold cross-validation.

Cross validation folds	% Classification on SNV preprocess data
1	75
2	95
3	90
4	75
5	80
6	90
7	81
8	75
9	81
10	85
Average	83

From the results of the tenfold cross-validation method, the average classification rate is found to vary from 75% to 95% and the average accuracy is obtained as 83% with SNV preprocessing. This result is quite encouraging, even though the data set is quite small with respect to the number of classes.

## Conclusion

In this paper, a methodology has been proposed to grade *Andrographis paniculata* samples using NIR spectroscopy and SVM classifier based on the content of marker molecules, andrographolides. Relative accuracies of estimating the andrographolides by NIR spectra of methanol extracts of the samples, and powdered leaf samples were compared taking the estimates obtained from HPLC analysis as the standard. The accuracy of estimation based on extracts was a little higher than the powder leaf samples. But it did not change the grading pattern of the samples. Support vector machine was used to grade the samples into three classes–Class I (best quality), Class II (intermediate quality) and Class III (poor quality). The average classification accuracy of tenfold cross validation of SVM was obtained as 83%. Thus, NIR based estimation of powdered leaf samples combined with SVM classifier can be a low-cost solution to grade the samples rapidly. A small range portable NIR instrument would serve as a field-lab deployable instrument for gradation of *Andrographis paniculata* samples by the industry.

## Data Availability

The raw data supporting the conclusions of this article will be made available by the authors, without undue reservation.
